# Density functional theory study of mechanical, thermal, and thermodynamic properties of zinc-blende CdS and CdSe

**DOI:** 10.1038/s41598-025-26168-w

**Published:** 2025-11-07

**Authors:** Teshome Gerbaba Edossa

**Affiliations:** Department of Physics, College of Natural & Computational Sciences, Wachemo University, Hosanna, 1000 Central Ethiopia Ethiopia

**Keywords:** DFT, DFT+U, ZB-CdS, ZB-CdSe, Elastic properties, Thermal and Thermodynamic properties, Chemistry, Materials science, Physics

## Abstract

This study investigates the mechanical, thermal, and thermodynamic properties of zinc-blende (zb) CdS and CdSe using Density Functional Theory (DFT) within the LDA, PBE, and PBE+U approximations. All three functionals confirm the mechanical stability of both compounds, with PBE+U providing results that best align with available theoretical and experimental data. Based on PBE+U calculations, CdS exhibits higher stiffness (B = 71.75 GPa, E = 36.71 GPa, G = 12.99 GPa) and faster sound velocity ($\nu$= 1828 $\hbox {ms}^{-1}$) than CdSe (B = 53.85 GPa, E = 38.88 GPa, G = 14.13 GPa, $\nu$ = 1746 $\hbox {ms}^{-1}$). Temperature-dependent analyses using the quasi-harmonic approximation reveal anomalous thermal contraction at low temperatures, transitioning to normal expansion beyond the zero thermal expansion points (113.92 K for CdS and 61.50 K for CdSe). The electron chemical potential shows a non-monotonic temperature dependence with transition temperatures of 1483 K for CdS and 853 K for CdSe. Heat capacities approach the Dulong-Petit limit ($\approx$ 49 J $\hbox {mol}^{-1}$ $\hbox {K}^{-1}$) at high temperatures, with CdSe reaching this limit earlier due to its softer lattice. CdSe also displays higher entropy, consistent with its heavier atomic mass and enhanced anharmonicity. Overall, CdS is mechanically stiffer and thermally more stable, while CdSe exhibits greater vibrational disorder. Overall, CdS is mechanically stiffer and thermally more stable, while CdSe shows greater anharmonicity and entropy.

## Introduction

Cadmium chalcogenides (CdX, where X represents sulfur, selenium, and tellurium) are a significant class of semiconductor materials. These materials have garnered substantial research interest due to their superior optoelectronic characteristics related to light interaction and electronic signal processing^1,6^. This versatility includes efficient light absorption, light emission, and the conversion of light into electricity (and vice versa)! This makes them essential building blocks for a wide array of technologies, from solar cells that power our homes to highly sensitive photodetectors that capture faint light signals, and even for cutting-edge applications like bioimaging, where we use light to see inside living tissues, and quantum computing, which promises to revolutionize computation^1,6^. For the effective design, optimization, and long-term performance and stability of devices utilizing these materials, a comprehensive understanding of their mechanical, thermal, and thermodynamic properties is indispensable. The response of these materials to varying temperatures and mechanical stresses directly dictates device reliability and operational efficiency.

Cadmium chalcogenides are polymorphic, meaning they can arrange their atoms in more than one way, resulting in different crystal structures^4^. The two main ways they like to organize themselves are into the hexagonal wurtzite structure, which is the most common and stable arrangement, and the cubic zinc-blende structure, which is a bit less common and not as energetically favorable. While wurtzite is the typical form we find, the zinc-blende structure has some unique advantages. One key advantage is that it’s isotropic, meaning its properties are the same in all directions. This uniformity, along with its particular electronic band structure (which dictates how electrons move within the material), makes it especially appealing for very specific applications, such as in microelectronics, where we need precise control over electron flow, and in nonlinear optics, where materials are used to manipulate light in sophisticated ways^6–8^.

For the effective design, optimization, and long-term performance and stability of devices utilizing these materials, a comprehensive understanding of their mechanical, thermal, and thermodynamic properties is indispensable^9,10^. Mechanical properties, such as elastic moduli, hardness, and fracture toughness, are crucial because they directly impact device durability and resistance to operational stresses^11,12^. If a material is too brittle or too soft, it won’t last long in a real-world application^11,12^. Similarly, Thermal properties, on the other hand, govern how a material behaves when it’s heated up. Thermal properties, including thermal conductivity and thermal expansion, govern heat dissipation and dimensional stability, which are critical for maintaining device reliability when temperatures fluctuate^11,12^.

Despite existing theoretical work, a comprehensive and internally consistent predictive study on the combined mechanical, thermal, and thermodynamic properties of zb-CdS and zb-CdSe, especially one that systematically compares both their mechanical and thermal properties in a systematic way and compares the effect of DFT functional choice and includes full temperature dependence is lacking^5,6,11^. Addressing this, the aim of the present work is twofold: first, to determine the most accurate theoretical framework by comparing LDA, PBE, and PBE+U approximations and explicitly examining the role of p-d coupling and Band Gap prediction. Second, we aim to calculate, compare, and contrast the full suite of mechanical stability constants, the zero thermal expansion (ZTE) points, and key thermodynamic quantities (entropy, heat capacities) for both CdS and CdSe across a wide temperature range using the validated PBE+U approach alongside the Quasi-Harmonic Approximation (QHA). These detailed, predictive results serve to establish CdS and CdSe as viable and stable candidates for specific technological applications in photoelectronics, sensing, and thermoelectrics^5,6,12,13^.

Therefore, this research is designed to fill that gap. We aim to conduct a detailed comparative analysis of the mechanical and thermal properties of zinc-blende CdS, and CdSe.

### Computational methods

Solving many-body problems involving interacting electrons poses a significant challenge, as analytical solutions are generally unattainable for systems with three or more interacting electrons^14,15^. It arises from mapping the complex interacting many-body electron problem onto a fictitious system of non-interacting electrons that reproduce the same ground state density. To overcome this, Density Functional Theory (DFT) has become a widely adopted approach in recent years^15,17^. DFT directly shifting the focus from the complex N-electron wavefunction, which depends on 3N spatial, coordinates, to the electron density, which depends on only three spatial coordinates (r). This fundamental shift is rooted in the Hohenberg-Kohn theorems, which provide the theoretical basis for DFT^14,17^.

In this study, all calculations were performed using the Quantum-ESPRESSO (QE) package, which implements the plane-wave pseudopotential method^16,17^. A key challenge in solving the Kohn-Sham equations within Density Functional Theory (DFT) lies in the approximation of the exchange-correlation (XC) potential, a complex term that accounts for many-body interactions. To address this, we employed several XC functional approximations such that the Local Density Approximation (LDA)^18^, the Perdew-Burke-Ernzerhof (PBE) Generalized Gradient Approximation (GGA)^19^, and GGA+U^20^.

Convergence tests for total energy with respect to plane-wave cutoff and k-point sampling were performed to ensure an energy accuracy. For zb-CdS, total energy convergence was achieved using 5 $\times$ 5 $\times$ 5 and 6 $\times$ 6 $\times$ 6 k-point grids for DFT + U and LDA/PBE, respectively, with a cutoff energy of 60 Ry. Specifically, convergence occurred at 55 Ry for LDA and PBE, and at 60 Ry for DFT + U. For zb-CdSe, total energy convergence was obtained with 7 $\times$ 7 $\times$ 7 (LDA, PBE, PBE0) and 6 $\times$ 6 $\times$ 6 (DFT + U) k-point grids, and a cutoff energy of 60 Ry for all approximations.

For this approach, the interaction between core and valence electrons was treated using the Projector Augmented-Wave (PAW) pseudopotential. In the pseudopotential approach, the chemically inert core electrons are effectively “frozen,” and only the valence electrons, which participate in chemical bonding, are explicitly considered. For Cadmium (Cd, atomic number 48), the valence electron configuration is [Kr] 4$\hbox {d}^{10}$ 5$\hbox {s}^2$, and for Sulfur (S, atomic number 16), it is [Ne] 3$\hbox {s}^2$ 3$\hbox {p}^4$.

In these calculations, the experimentally determined lattice parameters of CdS were used as the starting point. To ensure the reliability of our results, convergence tests were performed with respect to the electronic wave function cutoff energy and the k-point sampling of the Brillouin zone, which was generated using the Monkhorst-Pack scheme^21^. The cutoff energy for the plane-wave basis set was systematically increased until the total energy of the system converged to within 0.01 eV. Similarly, the density of the k-point mesh was increased until energy convergence was achieved. Following these convergence tests, A single-shot calculation was performed to determine the appropriate value of the Hubbard U parameter for the Cd 4d-orbitals. The calculated value was determined to be U=7.6 eV using the Linear Response method, consistent with previous literature studies on Cd-chalcogenides^46^ for Cd within the CdS system and 7 eV for Cd within the CdSe system. The Hubbard correction (PBE + U) was applied to the Cd 4d orbitals to correct p-d hybridization effects. This localization shifts the Cd 4d states deeper into the valence band, reducing p-d repulsion and thereby widening the band gap and improving structural properties in agreement with experiment^48^.

## Results

### Elastic properties of ZB-CdS and ZB-CdSe

Mechanical properties play a great role in the design and application of materials. The mechanical properties of a material such as fracture strength, Young’s modulus, shear modulus, Malleability, Ductility, Toughness, Resilience, Brittleness, Stiffness (Modulus of Elasticity/Young’s Modulus), Fatigue, Creep, Poisson’s Ratio and hardness determine the capability of a material to resist external or internal stresses [22-27]. The study of the mechanical behavior of ZB-CdX is useful to identify its various technological applications. Hence, the mechanical properties of ZB-CdX are based on its elastic constants, which indicate response to external forces. Therefore, the elastic constants of ZB-CdX are important parameters of a material and can provide valuable information about the mechanical stability, bonding character between adjacent atomic planes, brittleness, ductility, stiffness, and anisotropic character^28^. There are six components of stress and a corresponding six components of strain for the general 3-D case. Thus, Hooke’s law may be expressed as follows:

1 $$\begin{aligned} \delta _i = C_{ij}\epsilon _j \end{aligned}$$

2 $$\begin{aligned} \epsilon _j = S_{ij}\delta _j \end{aligned}$$

Using Density Functional Theory (DFT) with the LDA, PBE and PBE+U the elastic constant matrix for Zinc Blende Cadmium Chalcogenide (ZB-CdX) was calculated, revealing its fundamental mechanical response. The independent elastic constants were determined to be $\hbox {C}_{{11}}$, $\hbox {C}_{{12}}$, and $\hbox {C}_{{44}}$ was calculated as in Table 1.

**Table 1:** Calculated Independent Elastic Constants $C_{ij}$ (in GPa) for Zinc-blende $\text {CdS}$ and $\text {CdSe}$ compared with previous theoretical and experimental results.

Materials	Methods	$C_{11}$ (GPa)	$C_{12}$ (GPa)	$C_{44}$ (GPa)
CdS	LDA	66.86	45.60	26.53
	PBE	70.49	53.98	23.07
	PBE+U	80.41	67.42	20.60
	References (Other Theory)	66.8^37^, 70.0^36^	45.4^37^, 53.6^36^	26.6^37^, 27.2^36^
	References (Exp.)	N/A	N/A	N/A
CdSe	LDA	55.18	32.01	25.06
	PBE	57.61	39.78	21.97
	PBE+U	62.37	49.58	23.85
	References (Other Theory)	55.0^34^, 57.6^35^	32.0^34^, 39.7^35^	25.0^34^, 21.9^35^
	References (Exp.)	N/A	N/A	N/A

For zinc blend (ZB) crystal systems, the elastic constant tensor is characterized by three independent non-zero components: $\hbox {C}_{{11}}$, $\hbox {C}_{{12}}$, and $\hbox {C}_{{44}}$. The mechanical stability of such a crystal is fundamentally governed by Born-Huang’s lattice dynamic theory, which postulates that a stable crystal’s elastic constant matrix must be positive definite, implying that any infinitesimal deformation from equilibrium results in an increase in its total energy. For ZB crystals, these stability criteria translate into three essential conditions for the independent elastic constants: $\hbox {C}_{11} > 0$ , ensuring stability against uniaxial stress, where a negative value would lead to spontaneous collapse or expansion; $\hbox {C}_{{11}}$ - $\hbox {C}_{12} > 0$ , which guarantees stability against tetragonal shear deformation along the [110] direction on the (110) plane, with a violation implying a spontaneous transformation to a tetragonal structure; and $\hbox {C}_{44} > 0$ , guaranteeing stability against pure shear deformation on the (100) planes along the [010] direction, where a negative value would cause spontaneous lattice distortion [29 - 31]. Based on my data in Table 1, it is evident that for both materials and in all approximations, these three conditions are satisfied. This confirms the mechanical stability of the ZB-CdX in their respective Zinc Blende or cubic (fcc) phases under the specified approximations. From these independent elastic constants, we can derive other important macroscopic mechanical properties that provide further insight into the material’s behavior: Bulk Modulus (B) Resistance to volume change under hydrostatic pressure.

3 $$\begin{aligned} B = \frac{C_{11} + 2C_{12}}{3} \end{aligned}$$

Shear Modulus (G - Hill Average): Resistance to shape change (shear deformation).

$$\begin{aligned} G_V = \frac{C_{11}-C_{12}+3C_{44}}{5} \\ G_R = \frac{5(C_{11}-C_{12})C_{44}}{4C_{44}+3(C_{11}-C_{12})} \end{aligned}$$

4 $$\begin{aligned} G_H = \frac{G_V + G_H}{2} \end{aligned}$$

Young’s Modulus (E - Hill Average): Stiffness under uniaxial stress.

5 $$\begin{aligned} E = \frac{9BG_H}{3B+G_H} \end{aligned}$$

Poisson ratio ( $\nu$ - Hill average): Ratio of transverse strain to axial strain.

6 $$\begin{aligned} \nu = \frac{3B-2G_H}{2(3B+G_H)} \end{aligned}$$

Anisotropy Factor): If A $\ne$ 1, is an elastically anisotropic material, its elastic properties will vary depending on the crystallographic direction along which force is applied.

7 $$\begin{aligned} A = \frac{2C_{44}}{C_{11} - C_{12}} \end{aligned}$$

The Debye temperature ($\theta _D$) is a characteristic temperature of a solid, representing the maximum frequency of lattice vibrations (phonons) that can exist in the material.

8 $$\begin{aligned} \theta _D = \frac{h}{k_B}\left( \frac{3nN_A\rho }{4\pi M}\right) ^{1/3}{\bar{v}} \end{aligned}$$

Where h is Planck’s constant, $k_B$ is Boltzmann’s constant, n is the number of atoms per formula unit $N_A$ is Avogadro’s number, $\rho$ is the density of the material, M is the molar mass and ${\bar{v}}$ is the average Debye sound velocity

9 $$\begin{aligned} {\bar{v}} = \left[ \frac{1}{2}\left( \frac{1}{V_2^3}+\frac{1}{V_l^3}\right) \right] ^{-1/3} \end{aligned}$$

Where $v_s$ is the average shear wave velocity, $v_l$ = $V_p$ (Compressional Velocity) which is the speed of longitudinal waves (sound) in the material, indicating how fast compression and rarefaction propagate which is given by,

10 $$\begin{aligned} v_l = V_p = \sqrt{\frac{B+\frac{1}{3}G}{\rho }} \end{aligned}$$

Shear Wave Velocity ($v_s$ or $V_G$): This is the velocity of transverse waves, which involve shape changes without volume changes.

11 $$\begin{aligned} V_G = v_s = \sqrt{\frac{G}{\rho }} \end{aligned}$$

Where B is Bulk Modulus, G is Shear Modulus and $\rho$ represents the density of the material. Accordingly, I calculated that B. G, E, $\nu$, A, $\theta _D$, $\acute{v}$, $V_p$, and $v_s$ for ZB-CdX as shown in Table 2.

**Table 2:** Mechanical parameters of ZB-CdS and CdSe.

Materials	Methods	B(GPa)	E(GPa)	G(GPa)	$\nu$	A	$\theta _D$	${\bar{v}}(m/s)$	$V_p (m/s)$	$v_s(m/s)$
CdS	LDA	52.69	49.35	18.38	0.343	2.50	216.68	2157.03	3984.50	1944.29
	PBE	59.48	42.19	15.28	0.380	2.80	198.41	1975.15	4052.75	1773.01
	PBE+U	71.75	36.71	12.99	0.413	3.09	183.59	1827.57	4280.07	1634.67
	$\hbox {Theorys}^{ [36], [37]}$	$\sim$56.1	$\sim$42.1	$\sim$16.0	$\sim$0.380	$\sim$2.89	$\sim$190.1	N/A	$\sim$4022	$\sim$1740
	Exp.$^{ [33]}$	$\sim$63	N/A	N/A	N/A	N/A	190-250	N/A	N/A	N/A
CdSe	LDA	39.73	47.76	18.39	0.299	2.16	191.96	1995.37	3373.86	1804.96
	PBE	45.72	41.26	15.30	0.348	2.46	175.96	1829.08	3422.74	1646.40
	PBE+U	53.85	38.88	14.13	0.375	3.73	167.93	1745.58	3588.73	1582.50
	$\hbox {Theorys}^{ [35]}$	$\sim$45.4	$\sim$41.2	$\sim$15.3	$\sim$0.347	$\sim$2.45	$\sim$176.7	N/A	$\sim$3418	$\sim$1640
	Exp.$^{ [34]}$	$\sim$53-56	N/A	N/A	N/A	N/A	N/A	N/A	N/A	N/A

Table 2, the calculated bulk modulus (B), Young’s modulus (E), shear modulus (G), Poisson ratio ($\nu$), anisotropy factor (A), Debye temperature ($\theta _D$), average sound velocity (${\bar{v}}$), compressional wave velocity ($V_p$), and shear wave velocity ($V_s$) provide comprehensive insights into the mechanical behavior of these semiconductor materials. A consistent trend is observed across both CdS and CdSe as the approximation method changes from LDA to PBE, and then to PBE+U. For both materials, the transition from LDA to PBE and then to PBE+U.

As we can see in Table 2, CdS has a higher bulk modulus than CdSe in all approximations. This indicates that CdS is intrinsically more resistant to volumetric compression than CdSe. This is expected because of the larger atomic size of Se compared to that of S, leading to weaker bonds and lower stiffness in CdSe. In Table 2 again, CdS generally exhibits higher values of E and G compared to CdSe. This further supports the notion that CdS is mechanically stiffer than CdSe. CdS typically has a Poisson’s ratio higher than that of CdSe for the same approximation. This suggests that CdS might exhibit a slightly greater tendency toward lateral expansion/contraction under axial stress. The anisotropy factor is generally comparable, but CdSe (3.73 with PBE+U) shows a significantly higher anisotropy compared to CdS (3.09 with PBE+U), indicating that the directional dependence of elastic properties is more pronounced in CdSe, particularly under this correction. CdS consistently shows higher Debye temperatures and faster sound wave velocities (${\bar{v}}$, $V_p$ and $v_s$) across all methods compared to CdSe. This is a direct consequence of CdS being stiffer and having stronger interatomic bonds, leading to higher vibrational frequencies and faster wave propagation.

The calculated PBE values show remarkable consistency with other reported theoretical results for both CdS and CdSe, particularly for the Bulk Modulus, Young’s Modulus, Shear Modulus,Poisson’s Ratio, Anisotropy Factor, and Debye Temperature. For instance, the PBE Bulk Modulus for CdS (59.48 GPa) is close to other theoretical predictions around 56-57 GPa, and for CdSe (45.72 GPa), it aligns well with other values near 45 GPa. Where experimental data are available (primarily for Bulk Modulus), the PBE functional, and especially PBE+U for CdS’s Bulk Modulus, tend to provide values that are in reasonable agreement or slightly overestimate them, which is a known characteristic for some GGA-type functionals when compared to experimental values for bulk moduli. The consistency across different DFT methods and agreement with existing literature strengthens the reliability of the presented theoretical calculations for predicting the elastic behavior of ZB-CdS and ZB-CdSe.

The analysis of elastic properties for ZB-CdS and ZB-CdSe, using DFT with various functionals, confirms their mechanical stability. ZB-CdS is consistently stiffer and transmits sound faster than ZB-CdSe due to stronger bonding. Both materials exhibit elastic anisotropy and tend towards ductile behavior. The choice of functional impacts predicted values, but overall results align well with other theoretical findings, validating the calculations and providing crucial insights for material applications.

### Electronic structure and functional validation

The rigorous selection and validation of the DFT functional, a critical precursor to the QHA calculations, has been comprehensively documented in our prior works concerning zb-CdS^50^ and zb-CdSe^51^. These studies performed an exhaustive comparative analysis of the electronic structure, including the calculated Band Gap using LDA, PBE, PBE+U and PBE0 methods against experimental values. The standard LDA and PBE approximations were found to severely underestimate due to their inability to correctly describe the localized Cd d-states and the resulting spurious p-d hybridization. Through this validation process, the PBE+U and PBE0 method was determined to be the most accurate, as it successfully corrects this artifact, leading to values 2.45 eV - PBE+U and 2.48 eV - PBE0 for CdS and 1.83 eV and 1.94 eV for CdSe that are in strong agreement with experimental findings. Therefore, based on the superior predictive accuracy demonstrated in these dedicated electronic structure investigations, all subsequent calculations for the mechanical, thermal, and thermodynamic properties within the Quasi-Harmonic Approximation (QHA) in the present study utilize the validated PBE+U functional.

### Thermal and thermodynamic properties of ZB-CdS and ZB-CdSe)

Understanding the thermal and thermodynamic properties of ZB-CdS and ZB-CdSe moves beyond just their mechanical integrity to how they perform in environments where temperature fluctuations are key. These properties dictate fundamental aspects of material response, from their ability to store energy to their dimensional stability, and even their longevity in service. For materials like CdS and CdSe, which are widely used in optoelectronics, solar cells, thermoelectric devices, and various sensing applications, managing heat is not merely an engineering detail but a critical determinant of device efficiency, reliability, and lifespan. Predicting or measuring these thermal characteristics provides essential guidance for design optimization, ensuring the materials can withstand operational temperatures, dissipate excess heat effectively, and maintain stable performance under diverse thermal conditions^35–40^.

Based on this, properties such as vibrational free energy, electron chemical potential, entropy, and heat capacity (isochoric and isobaric), Grüneisen parameters, average Grüneisen parameters as a function of temperature, thermal expansion, and bulk moduli as a function of temperature, and zero-point energy were computed for ZB-CdS and ZB-CdSe using the Quasi-Harmonic Approximation (QHA). The QHA is the standard DFT-based approach to calculate temperature-dependent thermodynamic properties^38–43^. Vibrational Free Energy ($F_{vib}(V,T)$ or Helmholtz Free Energy) is given as (12)

12 $$\begin{aligned} F_{vib}(V,T) = \sum _{qj}\left[ \frac{1}{2}(\hbar \omega _{qj}(V) +k_BTlm\left( 1- e^{\frac{\hbar \omega _{qj}(V)}{k_BT}}\right) \right] \end{aligned}$$

Where $\hbar$ is the reduced Planck constant, $\omega _{qj}(V)$ is the frequency of the phonon mode with wave vector q and branch j at volume V, (These are obtained from harmonic phonon calculations), $k_B$ is the Boltzmann constant, The first term, $\frac{1}{2}\hbar \omega _{qj}(V)$, represents the zero-point energy (ZPE) contribution for each mode, The sum is over all phonon modes in the Brillouin zone. In practice, this is done by summing over a dense q-point mesh. Vibrational Energy ($E_{vib}(V, T)$ is derived from the vibrational free energy as (13)

13 $$\begin{aligned} E_{vib}(V,T) = F_{vib}(V,T)-T\left( \frac{\partial F_{vib}}{\partial T}\right) _V = \sum _{qj}\left[ \frac{1}{2}(\hbar \omega _{qj}(V) + \frac{\hbar \omega _{qj}}{e^{\frac{\hbar \omega _{qj}(V)}{k_BT}}-1}\right] \end{aligned}$$

Vibrational Entropy ($S_{vib}(V, T)$: Derived from the vibrational free energy as (14)

14 $$\begin{aligned} S_{vib}(V,T) = F_{vib}(V,T)-T\left( \frac{\partial F_{vib}}{\partial T}\right) _V = \sum _{qj}\left[ k_Bln\left( \frac{1}{1-e^{\frac{\hbar \omega _{qj}(V)}{k_BT}}}\right) - \frac{\hbar \omega _{qj}(V)}{T\left( e^{\frac{\hbar \omega _{qj}}{k_BT}-1} \right) }\right] \end{aligned}$$

Isochoric Heat Capacity): Derived from vibrational energy as (15)

15 $$\begin{aligned} C_v(v,T) = \left( \frac{\partial F_{vib}}{\partial T}\right) _V = \sum _{qj}k_B\left( \frac{\hbar \omega _{qj}(V)}{2k_BT}\right) ^2 \frac{1}{sinh^2\left( \frac{\hbar \omega _{qj}(V)}{2k_BT}\right) } \end{aligned}$$

Isothermal Bulk Modulus ($B_T(T)$) : Measures resistance to compression at constant temperature as (16).

16 $$\begin{aligned} B_T(T) = -V\left( \frac{\partial P}{\partial V}\right) _T = V\left( \frac{\partial ^2F_{tot}(V,T)}{\partial V^2}\right) _T \end{aligned}$$

Grüneisen Parameter ($\gamma _{qj}(V))$: For each phonon mode also calculated as (17):

17 $$\begin{aligned} \gamma _{qj}(V)) = \frac{V}{\omega _{qj}(V)}\left( \frac{\partial \omega _{qj}(V)}{\partial V}\right) _T \end{aligned}$$

This requires calculating phonon frequencies at slightly varied volumes. Average Grüneisen Parameter (${\bar{\gamma }} (T)$) , A thermally averaged Grüneisen parameter calculated as (18):

18 $$\begin{aligned} {\bar{\gamma }}(T) = \frac{\sum _{qj}\gamma _{qj}C_V(qj,T)}{\sum _{qj}C_V(qj,T)} = \frac{\sum _{qj}\gamma _{qj}C_V(qj,T)}{C_V(qj,T)} \end{aligned}$$

Where $C_V(qj, T)$ is the contribution of a single mode to the isochoric heat capacity Isobaric Heat Capacity $(C_p (T))$ Heat capacity at constant pressure:

19 $$\begin{aligned} C_P(T) = C_v(T) + TV(T)\alpha _V(T)^2B_T(T) \end{aligned}$$

Where $\alpha _V(T)$ is the volumetric thermal expansion coefficient, derived from V(T) and equal to

20 $$\begin{aligned} \alpha _V(T) = \frac{1}{V(T)}\left( \frac{\partial V}{\partial T}\right) _P \end{aligned}$$

### Temperature dependence of electron chemical potential ($\mu$)

The electron chemical potential ($\mu$), often referred to as the Fermi level at finite temperatures, is a critical thermodynamic property that governs the occupation probability of electron states in a semiconductor across varying temperatures. It is defined by the Fermi-Dirac distribution function.

21 $$\begin{aligned} \mu (T) = \frac{E_c-E_v}{2}+\frac{3}{4}k_BTln\left( \frac{m_h^*}{m_e^*}\right) \end{aligned}$$

Where, $E_c$ and $E_v$ are the energies of the conduction band minimum and valence band maximum, respectively, which define the band gap $E_g = E_c - E_v$. $k_B$ is the Boltzmann constant, and $m_h^*$ and $m_e^*$ are the effective masses of holes and electrons, respectively. the electron chemical potential lies precisely at the mid-gap, $(E_c+E_v)/2$ As temperature increases, $\mu$ typically shifts away from the mid-gap. The direction of this shift depends on the ratio of the effective masses if $m_h^* > m_e^*$ , $\mu$ shifts towards the conduction band as temperature increases; conversely, if $m_h^* < m_e^*$ , it shifts towards the valence band^38–43^.

Unlike simplified models, an accurate determination of $\mu$ at finite temperatures relies on the Density of States (DOS), denoted as g(E), which represents the number of available electronic states per unit energy.

The electron (n) and hole (p) concentrations are mathematically expressed as integrals over the respective conduction ($g_c(E))$ and valence ($g_v (E)$) band DOS, weighted by the Fermi-Dirac distribution function,

22 $$\begin{aligned} f(E,T,\mu ) = \frac{1}{e^{\frac{E-\mu }{k_BT}}+1} \end{aligned}$$

Electron and hole concentration calculation in band $n = \int _{E_c}^{\infty }g_c.f(E,T,\mu )dE$ and $p = \int _{-\infty }^{E_v}g_c(E).(1-f(E,T,\mu )dE$ The chemical potential $\mu$ is then implicitly determined by solving the charge neutrality condition (e.g, n = p for an intrinsic semiconductor, or $n + N_A^- = p + N_D^+$ for doped materials). This numerical integration and iterative solution directly incorporate the full complexity of the electronic band structure through the calculated DOS, allowing for accurate predictions even in cases of non-parabolic bands or degenerate doping conditions^38–43^.

**Fig. 1:**
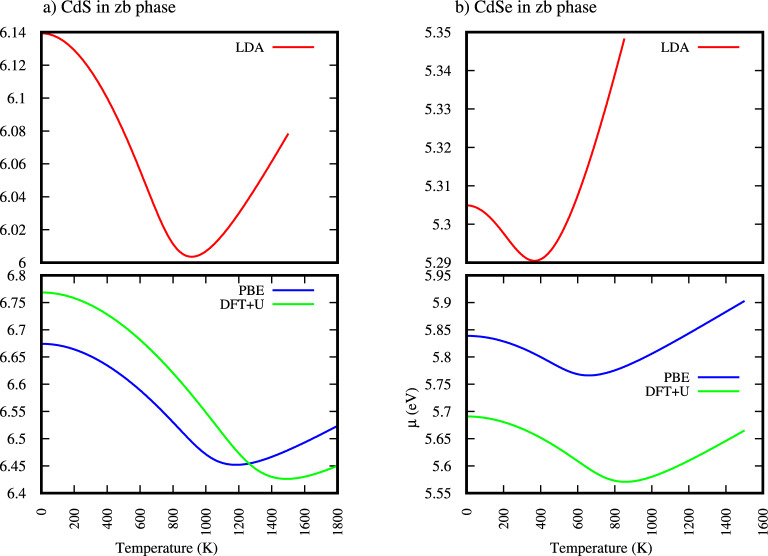
Electron chemical potential ($\mu$) as a function of temperature for (**a**) zb-CdS and (**b**) zb-CdSe crystals.

Based on this fact for ZB-CdS and ZB-CdSe, the calculated electron chemical potential as a function of temperature (Fig. 1) demonstrates a characteristic behavior. At low temperatures, $\mu$ initially decreases, reaching a minimum as shown in Fig. 1, up to temperature shown in Table 3 and Fig. 1, then starts to increasing again at higher temperatures. This is a non-monotonic behavior, and it is consistent with how intrinsic semiconductor behaves at high temperature^38–43^.

This tell us at low T Fermi level is near mid gap and as temperature increase more electron thermally excited Fermi level ($\mu$) shifts toward the conduction band ($m_h^* > m_e^*$ which is true for ZB-CdS and ZB-CdSe). After $\mu$ is reaching a minimum value after transition T, then at very high T the Fermi level tends to move back toward the center of the band gap, because the asymmetry in effective masses (which caused the shift at intermediate) becomes less significant and the entropy (disorder) from high carrier populations dominates. The system becomes more symmetric between electrons and holes ie carrier populations of electron in CB and hole in VB become more symmetric (nearly equal, significant consequence of their effective mass canceling out. This explains the U-shaped curve of Fig. 1:

**Table 3:** Calculated chemical potential ($\mu$ in eV) for zb- and zb-CdSe within different approximation at different temperature and Transition temperature T for zb- and zb-CdSe.

	$\mu$(at lower T = 1 k)			$\mu$(at high T =1500 k))			Transition T in k			Transition T in k		
Materials	LDA	PBE	PBE+U	LDA	PBE	PBE+U	LDA	PBE	PBE+U	LDA	PBE	PBE+U
CdS	6.14	6.67	6.77	6.00	6.45	6.43	6.078	6.524	6.427	910	1177	1483
CdSe	5.31	5.84	5.69	5.29	5.77	5.57	5.348	6.524	5.665	346	667	853

As depicted in Fig. 1, the electron chemical potential (Fermi level) for ZB-CdS exhibits a minimum point at specific temperature. This specific temperature, referred to as the transition temperature (Transition T) in Table 3, signifies a critical shift in the material’s electronic behavior. For ZB-CdS, Table 3 indicates this transition temperature is 910 for LDA, 1177 K for PBE and 1483 K for PBE+U functionals. Similarly, for ZB-CdSe, the Transition T is observed at lower temperatures, specifically 346 for LDA, 667 K for PBE and 853 K for PBE+U. At temperature where $\mu$ minimum point, the thermal generation of electron and hole populations reaches a state where they begin to balance out. Consequently, this transition temperature marks a shift from a regime where electron excitation might predominantly characterized by more symmetric carrier behavior, reflecting an increasingly intrinsic semiconductor response at higher temperatures. This balance is crucial for understanding high-temperature device operation and material stability for both ZB-CdS and ZB-CdSe.

Based on the data presented in Table 3, and Fig. 1 a notable difference in the transition behavior is observed between ZB-CdS and ZB-CdSe. ZB-CdS exhibits significantly higher transition temperatures. In contrast, ZB-CdSe reaches this transition point at considerably lower temperatures. This implies that ZB-CdSe achieves a balanced state of thermally generated electron and hole populations, characteristic of intrinsic semiconductor behavior, at much lower temperatures compared to ZB-CdS.

### Temperature dependence of heat capacity in zinc-blende CdS and CdSe

**Fig. 2:**
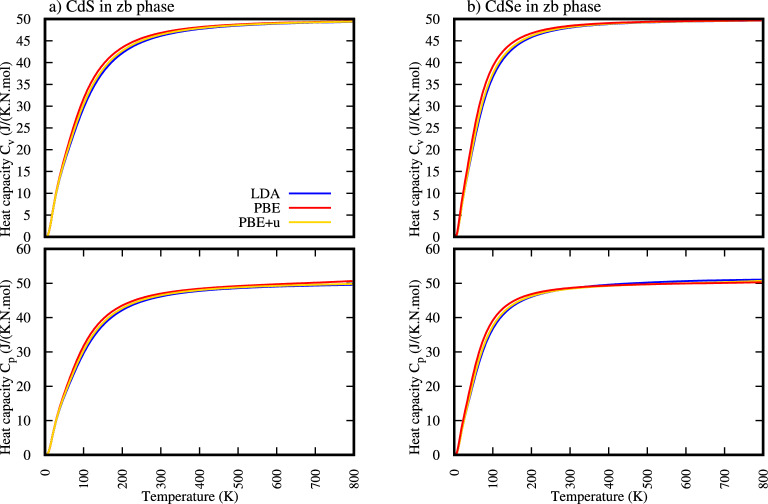
Temperature dependence of heat capacity: (**a**) zinc-blende CdS and (**b**) zinc-blende CdSe.

Figure 2, a critical component of an article discussing the thermal properties of II-VI semiconductors, meticulously illustrates the calculated isochoric heat Capacity (Cv) and isobaric heat Capacity (Cp) heat capacities for Cadmium Sulfide (CdS) and Cadmium Selenide (CdSe) in their zinc-blende phases across a temperature range up to 800 K. A critical point of this Fig. 2 lies in its demonstration of the convergence towards the Dulong-Petit limit at higher temperatures.

As temperature increases, both $C_v$ and $C_p$ for both CdS and CdSe exhibit a rapid increase from near zero, eventually saturating at 3R per atom beyond a certain temperature as shown in Fig. 2 and Table 4. The temperature beyond which the heat capacities ($C_v$ and $C_p$) of a solid converge towards the Dulong-Petit limit is often referred to in relation to the thermal Debye temperature ($\theta _d^{thermal}$).

**Table 4:** Calculated value of isochoric heat Capacity ($C_v$) and isobaric heat Capacity ($C_p$) of zink-bled CdS and CdSe.

	Heat capacity J/(K.N.mol)						Temperature limit ($\theta _D^{thermal}$) k					
	$C_V$			$C_P$			$C_V$			$C_P$		
Materials	LDA	PBE	PBE+U	LDA	PBE	PBE+U	LDA	PBE	PBE+U	LDA	PBE	PBE+U
CdS	47.40	48.60	8.33	48.60	49.13	48.87	496	466	454	505	490	514
CdSe	48.87	48.86	48.87	49.78	49.12	49.26	403	352	388	430	364	409

This saturation signifies that at these higher temperatures, all vibrational degrees of freedom are fully excited, and the classical equipartition theorem becomes applicable. Furthermore, the figure critically highlights the comparative performance of different Density Functional Theory (DFT) approximations, LDA, PBE, and PBE+U, in predicting these thermal properties. While all three methods show similar qualitative trends, subtle differences are observed, particularly in the saturation values and the rate of increase at lower temperatures, indicating the importance of selecting appropriate theoretical frameworks for accurate materials design and prediction. The consistent observation that $C_p$ is greater than $C_v$ across the entire temperature range, with the difference becoming more pronounced at higher temperatures, is also a critical and expected thermodynamic outcome, reflecting the work done against external pressure under isobaric conditions

From Table 4 and Fig. 2 the earlier convergence of Cadmium Selenide (CdSe) to the Dulong-Petit limit compared to Cadmium Sulfide (CdS), as seen in their heat capacity curves (,indicates that CdSe has a lower Debye temperature and a “softer” lattice with weaker average interatomic bonds. This difference primarily stems from the significantly heavier Selenium atom in CdSe compared to the Sulfur atom in CdS. Heavier atoms generally vibrate at lower frequencies, meaning less thermal energy is required to fully excite all vibrational modes in CdSe. Consequently, this suggests that CdSe’s lattice vibrations are easier to excite, leading to its heat capacity reaching the classical limit at a lower temperature, with potential implications for its thermal conductivity and anharmonic behavior at relatively lower temperatures.

### Temperature dependence bulk modulus and thermal expansion of zinc-blende CdS and CdSe

**Fig. 3:**
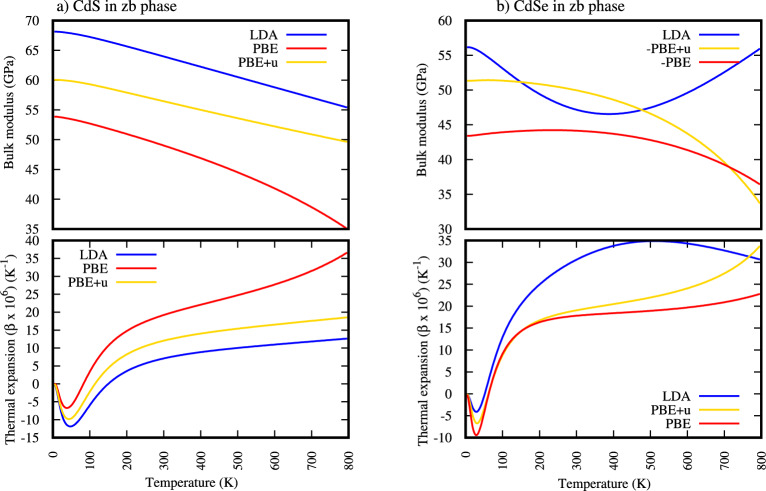
Temperature dependence of bulk modulus and thermal expansion in zinc-blende CdS and CdSe.

The Fig. 3 illustrates the temperature dependence of the bulk modulus for Zinc-Blende CdS and CdSe calculated using LDA, PBE, and PBE+U DFT functionals. A critical observation is the general thermal softening: the bulk modulus consistently decreases with increasing temperature, indicating reduced resistance to compression due to enhanced atomic vibrations and weakened interatomic forces from anharmonicity.

The Fig. 3 also clearly demonstrates the temperature dependence of thermal expansion, which arises from the anharmonic nature of the interatomic potential. The Fig. 3 shows as temperature begins to increase thermal expansion decrease reach minimum and then begin to increase is a slightly nuanced way of observing the specific features of of Zinc-Blende CdS and CdSe. This minimum point of therma expansion is referred to as peculiarity or anomaly and Its corresponding temperature is simply referred to as the temperature of maximum negative thermal expansion or transition temperature (transition T) .

At low temperatures, the thermal expansion coefficient of Zinc-Blende CdS is negative in temperature range of $\le$ 148 k LDA, $\le$ 82 k and $\le$ 112 k PBE+U and Similarly for Zinc-Blende CdSe thermal expansion is negative in temperature range of $\le$ 49 k LDA, $\le$ 61 k and $\le$ 61 k PBE+U. This negative Thermal Expansion (NTE) indicates that the material’s internal structure rearranges itself upon heating in a way that leads to an overall volume contraction. This often arises from specific phonon modes (lattice vibrations), such as “rigid unit modes” where polyhedra rotate or bond angles change, leading to a densification of the structure as thermal energy is added. It’s a complex phenomenon rooted in the material’s unique crystal structure and lattice dynamics. This means the material actually contracts upon heating in this temperature range, which is an unusual but well-documented phenomenon.

As the temperature increases from that minimum value of thermal expansion (maximum negative) shown in Table 5 and Fig. 3, the thermal expansion coefficient begins to increase (becomes less negative), eventually crossing zero and becoming positive.

**Table 5:** Temperature dependent critical points of thermal expansion for zb-CdS andCdSe.

	Peculiarity ($10^6$ /k)			(transition T) (k)			ZTE Temperature (k)		
Materials	LDA	PBE	PBE+U	LDA	PBE	PBE+U	LDA	PBE	PBE+U
CdS	-11.886	-6.747	-9.826	46	37	43	149.84	82.81	113.92
CdSe	-4.120	-9.475	-6.613	28	28	31	51.81	62.56	61.50

As seen in Table 5 and Fig. 3 the temperature at which the thermal expansion coefficient crosses zero ($\beta$ = 0) or (ZTE Temperature) is a critical point is calculated for both Zinc-Blende CdS and CdS in LDA, PBE AND PBE+U. At this precise temperature, the material’s volume remains essentially constant despite changes in temperature. Materials with a ZTE point are highly desirable for applications requiring extreme dimensional stability, such as precision instruments, aerospace components, and optical systems, as they minimize thermally induced stress and deformation. Table 5 and Fig. 3 the temperature at which the thermal expansion coefficient ($\beta$) crosses zero for CdS is greater than for CdSe, so we can conclude CdS maintains a state of either contraction or very low expansion up to a higher temperature compared to CdSe. This suggests that CdS exhibits better dimensional stability over a broader range of lower temperatures before its thermal expansion becomes significantly positive and CdSe will begin to expand or show a significant positive thermal expansion at a lower temperature than CdS. When the thermal expansion coefficient ($\beta$) crosses zero, it signifies a point of zero net volume change. Since this temperature is higher for CdS than for CdSe, it implies that CdS maintains better dimensional stability over a broader range of lower temperatures compared to CdSe.

The shift from NTE to PTE signifies that at higher temperatures, the more conventional anharmonic vibrations leading to bond stretching and overall expansion become dominant. This thermal expansion of both CdS and CdSe take place beyond ZTE Temperature as shown in Table 5 and Fig. 3.

This NTE behavior is a known feature in many zinc-blende semiconductors (e.g., Si, GaAs, ZnO) and is fundamentally related to the contributions of low-frequency transverse acoustic (TA) phonon modes, which possess negative mode-Grüneisen parameters in the low-frequency region^47,48^. This physical mechanism is fully consistent with our detailed mode-Grüneisen analysis presented in Section Grüneisen parameter as a function of temperature in CdS and CdSe

### Grüneisen parameter as a function of temperature in CdS and CdSe

This section investigates the temperature dependence of the Grüneisen parameter ($\gamma$) for zinc-blende (zb) CdS and CdSe, critical for understanding their thermomechanical properties. The Grüneisen parameter, a measure of anharmonicity, links the thermal expansion to the vibrational properties of a material. A positive $\gamma$ indicates normal thermal expansion, while a negative $\gamma$ suggests anomalous contraction.

**Fig. 4:**
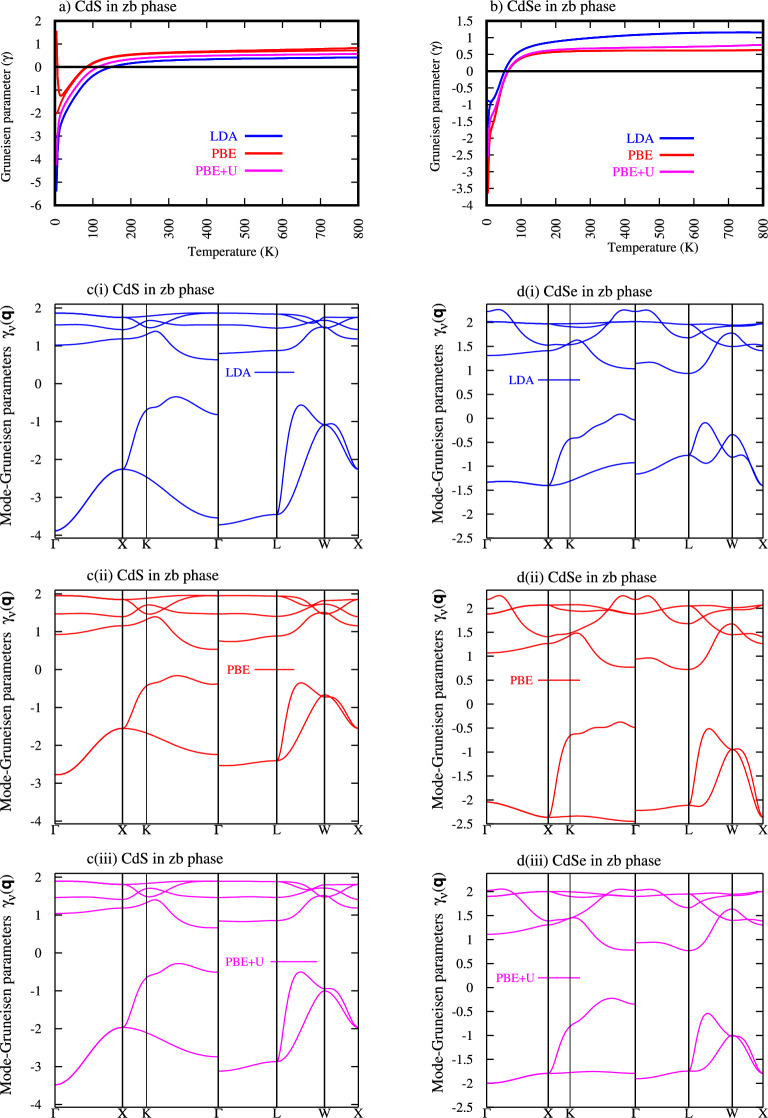
Grüneisen parameter as a function of temperature: (a) ZB-CdS, (b) ZB-CdSe, c(i) mode-Grüneisen parameter for ZB-CdS LDA, c(ii) mode-Grüneisen parameter for ZB-CdS PBE, c(iii) mode-Greisen parameter for ZB-CdS PBE+U and d(i) mode-Grüneisen parameter for ZB-CdSe LDA, d(ii) mode-Grüneisen parameter for ZB-CdSe PBE, d(iii) mode-Grüneisen parameter for ZB-CdSe.

Figure 4 a and b shows the Grüneisen parameter ($\gamma$) as a function of temperature (K) for zinc- blende (zb) phase: a) CdS and b) CdSe. Each plot presents results obtained using three different computational methods: LDA (blue line, PBE (red line), and PBE+U (magenta line).

From Fig. 4 a and b at very low temperatures (approaching $\theta$ K), $\gamma$ is significantly negative for both materials across all computational methods. This indicates that the dominant phonon modes at these temperatures contribute to anomalous thermal contraction. The magnitude of this negative value is more pronounced in CdS (reaching below -5.402 LDA, -2.039 PBE and -4.266 PBE+U) than in CdSe (reaching around -1.675 LDA, -3.5 PBE and -2.554)

As the temperature increases, $\gamma$ rapidly rises and transitions from negative to positive values. For CdS, this transition occurs at 149.84 K LDA, 82.81 K PBE, and 113.92 K PBE+U while for CdSe, is slightly earlier, around 51.81 K LDA, 62.56 K PBE, and 61.50 K PBE+U Table 5. This positive shift signifies the increasing dominance of phonon modes that contribute to normal thermal expansion as higher energy modes become populated

At higher temperatures (above $\sim$400 K) for CdS and above $\sim$300 K for CdSe, the Grüneisen parameter tends to saturate or show a much weaker temperature dependence. For both materials, $\gamma$ stabilizes at positive values, indicating normal thermal expansion at typical operating temperatures. The saturated value for CdS is generally higher (around 0.5-0.8) than for CdSe (around 0.7-1.2), depending on the method.

CdS generally exhibits more negative Grüneisen parameters at low temperatures and tends to saturate at higher positive values at high temperatures compared to CdSe. This suggests that the anharmonicity and its temperature evolution might be more pronounced in CdS. The transition from negative to positive $\gamma$ occurs at slightly lower temperatures for CdSe than for CdS,implying that the modes responsible for normal thermal expansion become dominant earlier in CdSe.

### Mode-Grüneisen parameters and anharmonicity in CdS and CdSe

Figure 4 c(i), c(ii), c(iii) and d(i), d(ii), d(iii) showing the mode-Grüneisen parameters ($\gamma _v(q)$) for CdS and CdSe in their zinc-blende (zb) phase, plotted along high-symmetry directions in the Brillouin zone with different approximation. The mode-Grüneisen parameter, unlike the macroscopic Grüneisen parameter, describes the response of individual phonon modes (characterized by their wave vector q and branch index v) to volume changes.

This section delves into the dispersion of mode-Grüneisen parameters ($\gamma _v(q)$) across the Brillouin zone for zinc-blende (zb) CdS and CdSe, providing a detailed insight into the anharmonic nature of their lattice vibrations. The mode-Grüneisen parameters reveal how individual phonon frequencies respond to changes in volume, directly influencing the overall thermal expansion behavior of the materials.

Figure 4 c and d, Both CdS and CdSe exhibit a wide range of mode-Grüneisen parameters, spanning from significantly negative values to positive values. This broad range indicates highly anisotropic and mode-dependent anharmonicity within the Brillouin zone . For both materials, the lower energy branches (corresponding to acoustic phonons, particularly near the $\Gamma$ point and extending towards $\chi$ and L points) predominantly show negative mode-Grüneisen parameters. This is a critical observation, as these negative values are responsible for the anomalous thermal contraction observed at low temperatures for these materials (as discussed in previous analyses of the macroscopic Grüneisen parameter). The magnitude of these negative values is significant, reaching below -4 for CdS and around -2.5 for CdSe, indicating a strong tendency for these modes to soften upon compression. Conversely, the higher energy branches (corresponding to optical phonons) generally exhibit positive mode-Grüneisen parameters. These modes contribute to normal thermal expansion at higher temperatures when they become significantly populated. The positive values often peak around 1.5 to 2.5, varying across different high-symmetry points.

CdS generally shows more negative mode-Grüneisen parameters in its acoustic branches compared to CdSe. This suggests that the anomalous low-temperature thermal contraction might be more pronounced or extend to a wider range of acoustic modes in CdS than in CdSe, aligning with the macroscopic Grüneisen parameter observations. While both materials show positive values for optical modes, the specific distribution and peak values vary. Overall, the range of ($\gamma _v(q)$) seems broader in CdS, indicating potentially stronger overall anharmonicity. Despite quantitative differences, the qualitative trends - negative values for lower energy modes and positive values for higher energy modes - are consistent between CdS and CdSe, reflecting their similar crystal structures and bonding characteristics.

### Vibrational energy and entropy of ZB-CdS and ZB-CdSe

**Fig. 5:**
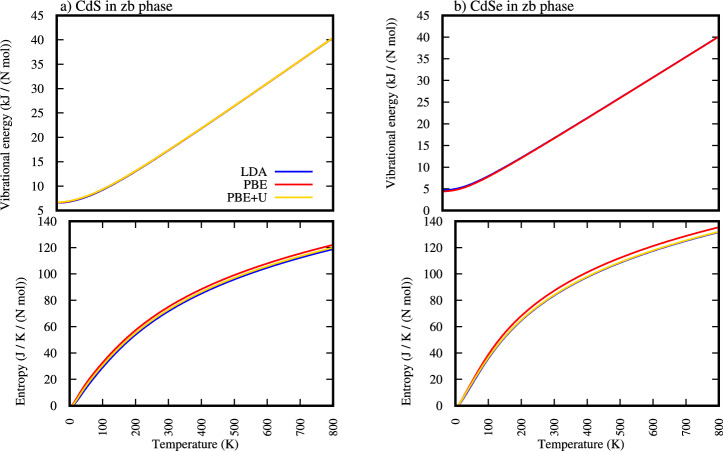
Vibrational energy and entropy of zinc-blende *CdS* and *CdSe*.

This section details the vibrational energy and entropy of zinc-blende (ZB) structured Cadmium Sulfide (CdS) and Cadmium Selenide (CdSe), as derived from different theoretical approximations: LDA, PBE and PBE+U. The top panels of Fig. 5 displays the vibrational energy as a function of temperature for both zinc-blende (ZB) structured Cadmium Sulfide (CdS) and Cadmium Selenide (CdSe), calculated using the LDA, PBE, and PBE+U approximations.

For both ZB-CdS and ZB-CdSe, the vibrational energy exhibits a consistent and significant increase with rising temperature [Fig. 5]. This is a characteristic behavior of solids, where increasing thermal energy excites more phonon modes, leading to higher average vibrational amplitudes and energies of the atoms in the lattice. At very low temperatures, the vibrational energy approaches a non-zero value [Table 6], representing the zero-point energy due to quantum mechanical effects. As the temperature rises, the vibrational energy increases almost linearly at higher temperatures, indicating the population of more phonon states.

**Table 6:** Calculated values of Vibrational energy of ZB-CdS and ZB-CdSe at very low temperatures (at 1.00K temperature).

Materials	CdS			CdSe		
Methods	LDA	PBE	PBE+U	LDA	PBE	PBE+U
Vibrational energy (KJ/cell)	6.596	6.651	6.718	4.731	4.437	4.817

Upon careful inspection of Fig. 5 and Table 6 the low-temperature region (approximately below $\sim$400 K), a subtle but distinct difference is observable: the vibrational energy of ZB-CdS appears to be slightly higher than that of ZB-CdSe. This initial difference can be attributed to the nature of zero-point energy. The zero-point energy of a system is inversely related to the atomic masses; lighter atoms (like sulfur in CdS) generally vibrate at higher frequencies, leading to a larger zero-point energy compared to systems with heavier atoms (like selenium in CdSe). Thus, at temperatures where only the lowest energy vibrational modes are excited and the zero-point energy forms a significant component of the total vibrational energy, CdS exhibits a marginally higher energy.

However, as the temperature increases, particularly above   400 K, the vibrational energy curves for ZB-CdS and ZB-CdSe become virtually indistinguishable, overlapping almost perfectly across the higher temperature range (up to 800 K). This convergence at higher temperatures indicates that the influence of the zero-point energy becomes less dominant as more and more vibrational modes across the entire phonon spectrum become thermally populated. At these higher temperatures, the overall thermal excitation energy effectively “smooths out” the initial mass-dependent differences in zero-point energy. The total energy stored in the vibrational modes over the entire spectrum becomes highly comparable for these two structurally similar compounds, suggesting that their overall lattice dynamics and energy absorption capabilities become very similar once a broad range of phonon states are excited.

The bottom panels of Fig. 5 displays entropy of both ZB-CdS and ZB-CdSe, the entropy increases monotonically with temperature. This is a fundamental thermodynamic behavior, as higher temperatures lead to a greater number of accessible microstates and thus increased disorder within the crystal lattice. At 0 K, the entropy is zero, consistent with a perfectly ordered crystalline state (assuming a pure, perfect crystal). As temperature rises, atoms vibrate with larger amplitudes and occupy a wider range of positions, leading to an increase in configurational and vibrational entropy.

**Fig. 6:**
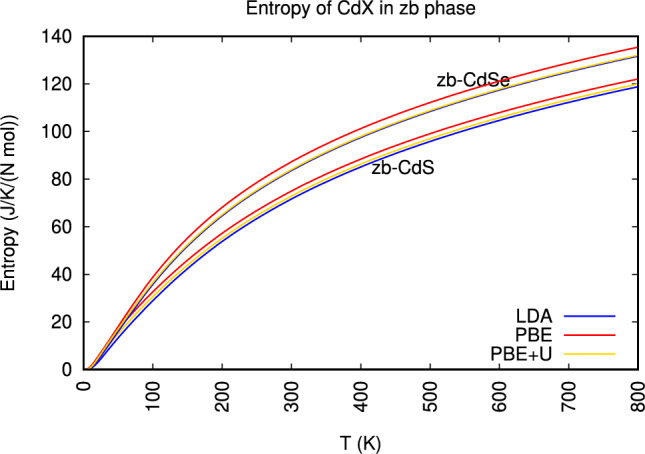
Comparison of entropy between zinc-blende *CdS* and *CdSe*.

Upon careful observation of Fig. 6 points that ZB-CdSe consistently exhibits higher entropy values than ZB-CdS at above temperature range  100 k up to 800 K. This difference is clearly visible, with the curves for ZB-CdSe lying above those for ZB-CdS .

This difference in entropy can be attributed primarily to the difference in the atomic mass of the anion. Selenium (Se) is significantly heavier than Sulfur (S). In general, heavier atoms lead to lower vibrational frequencies (softer phonons) and a greater density of states at lower energies. This means that a wider range of vibrational modes can be excited at a given temperature, contributing to higher vibrational entropy. The increased mass of the anion in CdSe, therefore, allows for a greater degree of thermal disorder compared to CdS at the same temperature.

## Conclusion

This theoretical study provides a comprehensive set of validated mechanical and thermal properties for zb-CdS and zb-CdSe using Density Functional Theory (DFT) across the LDA, PBE, and PBE+U approximations, combined with the Quasi-Harmonic Approximation (QHA). Based on the dedicated electronic structure validations performed in our prior works concerning CdS^50^ and CdSe^51^, the PBE+U method was confirmed as the most accurate. This method successfully corrects the spurious p-d hybridization, yielding highly accurate Band Gap values 2.45 eV - PBE+U and 2.48 eV - PBE0 for CdS^50^ and 1.83 eV and 1.94 eV for CdSe^51^ which validates its use for all derived properties. Quantitatively, CdS was found to be the stiffer material (Bulk Modulus B $\approx$ 71.75 Gpa) compared to Bulk Modulus B $\approx$ 53.85 GPa for CdSe. Thermally, both compounds exhibit anomalous Negative Thermal Expansion (NTE) at low temperatures; a phenomenon rooted in the negative mode-Grüneisen parameters of the TA phonons; with identifiable Zero Thermal Expansion (ZTE) points $\approx$ 113.92 K for zb-CdS and $\approx$ 61.50 K for zb-CdSe. These calculated results establish crucial design parameters: the validated band structure supports photoelectronic applications; the full set of elastic constants is key for sensing and MEMS devices; and the identified NTE and ZTE points are vital for achieving thermal stability in advanced thermoelectric composites.

## Data Availability

All data supporting the findings of this study are contained within the main manuscript. Additional raw data and processed outputs are available from the corresponding author upon reasonable request.

## Code availability

The codes and input files used for this study are available from the corresponding author upon reasonable request.
